# The impact of patients suicide on the mental health of psychotherapists

**DOI:** 10.1038/s41598-026-67919-7

**Published:** 2026-09-03

**Authors:** Annabelle Siebert, Thomas Schnell, Janina Lehmann, Rüdiger Nübling

**Affiliations:** 1 Medical School Hamburg, University of Applied Sciences and Medical University, Am Kaiserkai 1, 20457 Hamburg, Germany; 2 GfQG - Society for Quality in Healthcare, Wendtstr. 1, 76185 Karlsruhe, Germany; 3 Landespsychotherapeutenkammer Baden-Württemberg, Jägerstr. 40, 70174 Stuttgart, Germany

**Keywords:** Patient suicide, Clinician suicide loss, Mental health impact, Suicide postvention, Post-traumatic stress disorder, Health services, Public health, Therapeutics, Health care, Health occupations, Signs and symptoms

## Abstract

A great number of psychotherapists and psychiatrists experience at least one patient suicide during their career. This study aims to build upon previous research findings concerning the impact of experienced patient suicide on the mental health of practicing and trainee psychotherapists in a German sample. A central aspect involves examining the temporal development of perceived distress and identifying short- and long-term emotional effects of suicide through a mixed methods approach to derive corresponding implications for postvention. The online survey revealed relatively low levels of symptoms on the administered PTSD scale. Nevertheless, a subset of participants reported clinically significant symptoms of post-traumatic stress disorder (PTSD). Particularly intense feelings of grief, shock, and horror also play a prominent role. Suicide leads to psychotherapists experiencing fear when assessing suicidal patients. Factors that could influence the extent of the impact of suicide include the patient-therapist relationship, lack of support, perception of suicide as violent, and its unpredictability. These factors continue to require exploration to gain a comprehensive understanding of their effects following patient suicide.

**Supplementary Information:**

The online version contains supplementary material available at https://doi.org/10.1038/s41598-026-67919-7.

## Introduction

Suicidology, as a scientific field, focuses on suicidal behavior, epidemiology and causes of suicidality, and suicide prevention. Besides these core research areas, the consequences of suicide, especially for survivors, are also a key focus of suicide research. This includes the emotional impact on the bereaved, such as complex grief reactions and coping with loss. Tal Young and colleagues^1^ describe that losing a close person to suicide often leads to feelings of grief, guilt, confusion, rejection, shame, and anger. Furthermore, survivors are at an increased risk of developing depression and PTSD or exhibiting suicidal behaviors themselves. Additionally, survivors are more likely to develop a prolonged complex grief reaction^1^. Complex grief reaction involves a prolonged grieving process characterized by increased intensity and quality of grief, leading to significant impairments in various life areas due to grief symptoms^2^.

In a certain sense, psychotherapists and psychiatrists could be considered as survivors of patient suicides as well. A survey by Wurst and colleagues^3^ found that about 38% of psychiatry trainees, approximately 37% of senior psychiatrists, and nearly 23% of psychologists have experienced at least one patient suicide. The frequency of patient suicides experienced by psychotherapists and psychiatrists has been reported in numerous studies, with the numbers varying significantly depending on the study^4,5^. However, the exploration of the extent of the impact on the psychotherapist’s mental health and the intensity of perceived burden after a patient’s suicide might be even more important than how often a therapistexperience losing a patient by suicide. A quantitative study by Gibbons and colleagues^6^ examined the impact of completed patient suicides on mental health and clinical practice of psychiatrists. Respondents were further asked which resources they found helpful following the patient suicide and if they thought it could have been prevented. Key themes that concerned the respondents after the suicide, such as feelings of guilt and self-doubt, painful emotions (e.g. sadness, worry, anxiety and anger) and changes in clinical practice were identified^6^ and also consistent with previous studies^7^. Their questionnaire included free text questions as well, in which some respondents stated that they considered a change of career after the incident (27%). These reactions may develop into clinically significant mental disorders, such as depression and PTSD, particularly among psychotherapists with greater exposure to patient suicide^8^. In addition, many clinicians begin to question their therapeutic competence after such an event^9^. Those who experience multiple patient suicides appear to be especially vulnerable to psychological distress^10^.

Since psychiatrists and psychotherapists treat a large number of patients during their careers, there is a relatively high likelihood of experiencing a patient suicide^11^. In Germany, about one person dies by suicide every hour – while suicides account for about 1% of total deaths, it remains a taboo subject^12^. In psychiatric care facilities, up to 40% of admissions involve patients at risk of suicide, and suicides cannot always be prevented despite excellent communication, competence, and control^13^. Therefore, it is crucial to determine the extent to which mental health professionals are affected by the consequences of a patient suicide. This includes both the acute psychological burden and the short- and long-term emotional impacts, to derive how best to prepare those in these professions for such events. Another essential aspect could be support services, as the question arises as to which interventions are perceived as effective and how existing ones can be designed or expanded in the future. This could lead to important implications and development opportunities for postvention, or aftercare, for mental health professionals following a patient suicide.

## Aim of the present study

The subject of this work is the investigation of a relationship between an experienced patient suicide and the mental health of treating psychotherapists and psychiatrists. Additionally, it aims to explore the short- and long-term emotional impacts such a suicide has on psychotherapists. Furthermore, the study seeks to identify which postvention interventions have already been used and implemented and how helpful psychotherapists found them.

## Methods

### Study design

In this study a mixed-methods design was used, more specifically, a convergent parallel design. Quantitative and qualitative data were collected simultaneously, analyzed separately and finally integrated to compare findings or confirm results. The study was conducted by the Medical School Hamburg (MSH; Germany) and the State Psychotherapists Chamber of Baden-Württemberg (Stuttgart, Germany), carried out in the form of an online survey. The data were collected in anonymized written form via the online survey tool Unipark. Data collection took place in two phases (September–December 2023 and April–June 2024). The study participants were informed about the objectives of the study and the content of the questionnaire at the beginning of the survey. They were told that participation was voluntary and that the data would be collected anonymously, so no personal conclusions could be drawn. The participants were only able to complete the survey after they had read this information and given their consent. All procedures were in accordance with the latest revision of the declaration of Helsinki and were approved by the ethics committee of the MSH (ethics vote number: MSH-2024/318).

### Questionnaires

Currently, no validated instrument exists for assessing the impact of experiencing a patient suicide on psychotherapist’s mental health. Therefore, studies measuring related constructs or addressing similar questions were identified through a literature review. Portions of these questionnaires were modified and combined with already validated and widely used questionnaires like the Impact of Event Scale (IES-R^14^that is used to measure PTSD symptoms. The potential effects of patient suicide on therapists’ mental health were captured using the IES-R subscales of intrusion (Cronbachs α = 0.90), avoidance (α = 0.79), and hyperarousal (α = 0.90^15^). Participants were instructed to complete the IES-R twice: once retrospectively, based on the two-week period following the suicide (measurement option 1), and once more concerning their current thoughts and feelings related to the suicide during the past two weeks (measurement option 2). Accordingly, data were treated as if they were from two measurement points in the analysis. The items from the mentioned subscales were summed to subscale scores, which then produced an overall score for each participant^14^. Additionally, short- and long-term emotional impacts were measured using a questionnaire which was adopted from Kleespies and colleagues^16^. For short-term impacts, participants were asked to indicate how they felt immediately after the suicide on a 7-point Likert scale ranging from “completely applies” to “does not apply at all.” For long-term emotional impacts, participants were instructed to indicate to what extent certain emotions or thoughts had occurred in the context of their therapeutic work. This was also done on a 7-point Likert scale. Furthermore, the questionnaire included various postvention intervention strategies, which were to be evaluated for their usefulness in coping with the suicide (from “very helpful” to “not helpful at all” on a 5-point Likert scale).

### Participants and recruiting process

The study targets psychological as well as medical psychotherapists, including psychotherapists in training and child and adolescent psychotherapists who have personal experience with a patient´s suicide. These includes patients they have treated directly, as well as patients they have co-treated, for example, as part of an inpatient therapeutic team. The study employed a quantitative cross-sectional design, in which a one-time measurement was conducted via an online questionnaire (using UniPark survey software). Approximately 4,500 members of the Baden-Württemberg State Chamber of Psychotherapists (LPK BW), approximately 200 psychotherapy training institutes, as well as individual practices and clinics for psychiatry and psychotherapy in 9 federal states were contacted via email. At the time of the survey, there were approximately *n* = 9,000 trainees at the institutes who were already treating patients on an outpatient or inpatient basis as part of their training. At the institutes, information about the study was mostly available via bulletin boards; realistically, this meant that a maximum of 50% of the eligible training candidates could be reached through this channel. The number of psychotherapists reachable through hospitals and outpatient practices can be estimated at approximately 250–300. This means that the email reached a maximum of approximately 10,000 people. Assuming that approximately 25% have personally experienced a patient’s suicide, the population can be estimated at approximately 2,500, which corresponds to a response rate of about 5%. Data collection took place in cooperation with the Medical School Hamburg (MSH), the LPK BW, and the GfQG Karlsruhe. Completing the questionnaire took 15–20 min.

### Quantitative analysis

The quantitative data analysis was conducted using the statistical software IBM SPSS Statistics (IBM Corp. Version 27.0, Armonk, N.Y.). Descriptive statistics were produced for the collected variables. Since the data in both cases were not normally distributed according to the Shapiro-Wilk test, frequencies were also considered for the analysis. The IES-R was computed (a) as raw score scale (sum of the single items) and (b) using the formula given by Maercker & Schützwohl^14,15^.

$$IES - Scale{\text{ }} = {\text{ }}( - {\text{ }}0.02{\text{ }}*{\text{ }}Intrusion){\text{ }} + {\text{ }}(0.07{\text{ }}*{\text{ }}Avoidance){\text{ }} + {\text{ }}(0.15{\text{ }}*{\text{ }}Hyperarousal){\text{ }}$$

and evaluated using a paired t-test, as the assumptions for a t-test were met. Before conducting the paired t-tests, the dataset was checked for outliers. The individual subscales as well as the overall IES scores at the first and second measurement option were compared. The average time between the two measurement options was approximately 6.2 years (SD = 6,4; maximum: 32 years; minimum: 2 weeks).

### Exploratory qualitative data analysis

An exploratory data analysis was conducted to at least obtain an initial impression of the collected material, as the qualitative results could potentially complement those of the quantitative methods. The qualitative data analysis followed the structured content analysis procedure according to Mayring^17^. The free-text responses to the online survey item “Please specify the characteristics of the suicide that made it particularly impactful for you” were analyzed. The individual responses were imported into MAXQDA 2024^18^. The category formation was partially inductive and partially deductive. The starting point of the deductive approach was the work of Fairman and colleagues^19^, as the authors identified subcategories such as guilt, self-doubt, and grief. The qualitative analysis was conducted in an exploratory and supplementary manner, as the open-ended responses could provide information on psychological distress and the effects of the suicide on the treating professionals in addition to the overall IES-R scores. Furthermore, this approach allows the scores on the IES-R subscales to be contextualized and interpreted more comprehensively, as the IES-R questionnaire may not capture all relevant constructs that influence the mental health of the respondents. However, these could be identified by describing the characteristics that made this suicide particularly distressing for the respective clinician. For the analysis of the responses, the content-analytic structuring technique was chosen, in which categories are defined to be used for coding the material^17^. To this end, overarching categories were first established, which were then further refined over the course of the analysis. Anchor examples (typical text passages) were identified for the precisely defined categories. In addition, coding rules were established for each response category so that the units to be analysed could be assigned as unambiguously as possible. In many cases, an analysis unit could, in principle, have been classified into several categories, which is why the category that fit best was always chosen. Any disagreements that arose between the coders were resolved through discussion. In case this did not lead to a decision, a third coder was involved.

## Results

### Patient and therapist characteristics

A total of 130 out of 281 respondents completed the survey and met the inclusion criteria as described above. This corresponds to a completion rate of 46.26%. The remaining participants either did not complete the survey or had not experienced a patient’s suicide, so the survey ended when they answered “no” to that question. After data cleaning, a total of 117 respondents were included in the analysis. For sample characteristics regarding patients and therapists see Table 1. About half of the individuals who died by suicide were female (48.7%), most patients were between 19 and 30 years old (27.4%) or between 51 and 60 years old (23.1%). Two third of the patients (68.4%) were being personally treated by the respondent at the time of the suicide. Of these, about half were in inpatient care (52%), and almost as many were treated in an outpatient setting (44.3%). Nearly half of the suicides occurred in the patients’ home environment (44.4%) and about a third (29.1%) in public space, but 20 individuals (17.1%) died by suicide in the ward where they were being treated at the time of death, and nine patients did so elsewhere on the hospital grounds (7.7%). The most frequently chosen method was hanging (36.8%), closely followed by jumping (18.8% and medication or drug overdose (17.1%).

**Table 1:** Characteristics of the patients, location and type of suicide; characteristics of the therapists (*n* = 117).

Patients		*n*	Valid %
Age	14–18	10	8.5
	19–30	32	27.4
	31–40	13	11.1
	41–50	25	21.7
	51–60	27	23.1
	61–70	7	6.0
	> 70	3	2.6
Gender	Female	57	48.7
	Male	58	49.6
	Non-binary	2	1.7
Location of suicide	in the patient’s home environment	52	44.4
	public space (e.g. highway, railways, forest)	34	29.1
	in the hospital: station	20	17.1
	in the hospital: elsewhere	9	7.7
Personal treatment	yes	80	68.4
Type of suicide	hang	43	36.8
	medication/drugs	20	17.1
	jump	22	18.8
	Drowning	7	6.0
	suffocate	6	5.1
	other	19	16.2
**Therapists**			
Age	< 35	30	25.6
	36–40	29	24.8
	41–45	17	14.5
	46–50	15	12.8
	51–60	12	10.3
	> 60	14	12.0
Gender	Female	87	74.4
	Male	28	23.9
	Non-binary	2	1.7
Type of Therapist	Psychological Psychotherapist	71	60,7
	Medical Psychotherapist	16	13,7
	Child and Adolescent Psychotherapist	6	5,1
	Psychotherapist in Training	19	16,2
	Other	5	4,3
Work in psychiatry	Years	117	8.6/10.0
Work in psychotherapy	Years	117	11.2/9.5
Psychotherapy method	Cognitive Behavior Therapy	84	71.8
(multiple answers possible)	Psychoanalytic oriented Therapy	26	22.2
	Psychoanalysis	5	4.3
	Systemic Therapy	9	7.7
	other	4	3,4

Basic psychotherapist characteristics are shown in the lower half of Table 1. Slightly more than half of the mostly female (74.4%) psychotherapists who took part in the study were up to 40 years old, and only about a quarter were older than 50 years. The average working life in psychiatry was just under 9 years, and that in psychotherapy was just over 11 years. The predominantly practiced therapy method was CBT (71.8%), while psychoanalytic procedures were represented by just over a quarter.

### Attitudes towards suicidality

The number of experienced patient suicides ranged between 0 and 8, with 58.8% of respondents indicating that one of their patients had died by suicide, and 29.0% reporting two or three suicides, respectively (M = 1,96; SD = 1,68; Table 2). Regarding general views, most practitioners considered suicides to be partially predictable (73.5%) and partially preventable (65.0%; Table 3). In contrast, few psychotherapistsbelieved that suicides were not predictable at all (7.7%) or not preventable at all (5.1%). When respondents were asked to retrospectively assess the predictability and preventability of the suicide they found “most distressing,” 32.5% stated that the suicide of their own patients was not predictable at all, and 30.8% rated it as not preventable at all. Most respondents considered the experienced patient suicide to be somewhat predictable (53.0%) and somewhat preventable (54.7). A few psychotherapistsbelieved the suicide was largely preventable (7.7%) or largely predictable (10.3%).

**Table 2:** Number of experienced suicides (*n* = 117).

No. of suicides	Valid percentage (%)
1	58.8
2–3	29.0
≥ 4	11.2

M = 1.96 SD = 1.68, Min = 1/Max = 10.

**Table 3:** General and specific views on suicidality (*n* = 117).

	1	2	3	4	I don’t know	M	SD
Overall predictability	7.7	73.5	18.8	0	--	2.11	0.50
Overall preventability	5.1	65.0	29.1	0.9	--	2.25	0.56
Predictability most distressing Suicide	32.5	53.0	10.3	0.9	3.4	1.89	0.87
Preventability most distressing Suicide	30.8	54.7	7.7	0.9	6.0	1.96	0.98

Self-assessment was conducted on a 4-point Likert scale: 1 = “not at all”, 2 = “partially”/“somewhat”, 3 = “mostly”/“largely” to 4 = “completely”).

### Perceived helpfulness of interventions and support

**Fig. 1:**
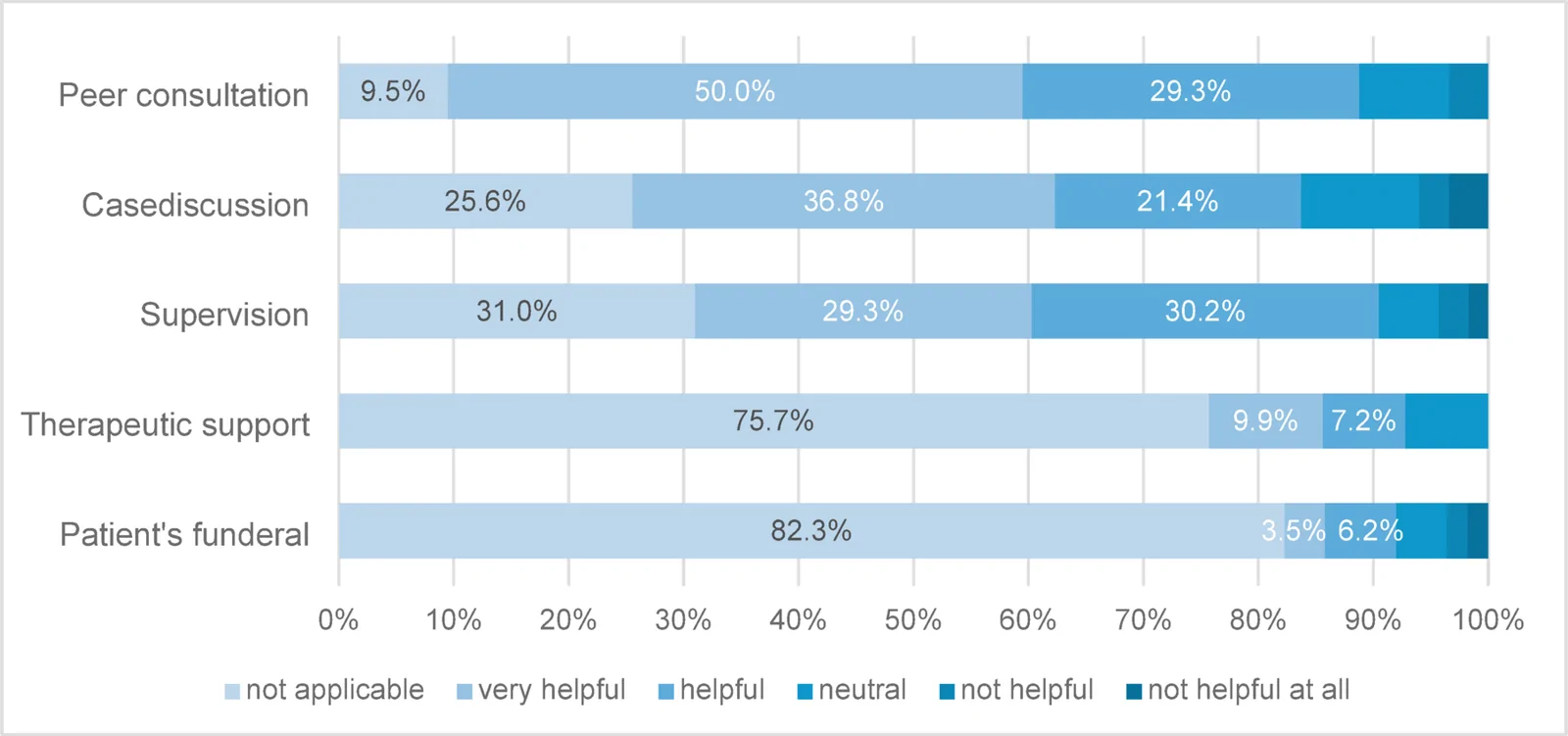
Perceived helpfulness of various interventions in coping regarding the suicide *Note*: 5-point Likert scale from 1="not helpful at all” to 5="very helpful” and an additional category “I don’t know,” with response frequencies in percentages (%); *n* = 117.

When supervision took place after the suicide, most respondents found it “very helpful” (29.3%) or “helpful” (30.2%) (Fig. 1). However, in 31.0% of cases, no supervision took place or was not utilized following the suicide. Peer consultations occurred more frequently overall and were also more frequently rated as very helpful (50%) or helpful (29.3%). Like supervision, case discussions did not occur in 25.6% of cases, but among those who conducted case discussions, the majority found them “very helpful” (36.8%) or “helpful” (21.4%). Only 17.7% of psychotherapistsattended the patient’s funeral, and the effectiveness of the funeral in coping with the suicide was perceived differently. Among respondents who attended the funeral, most of them found it helpful. Similar results were found among respondents who had received therapeutic support.

**Fig. 2:**
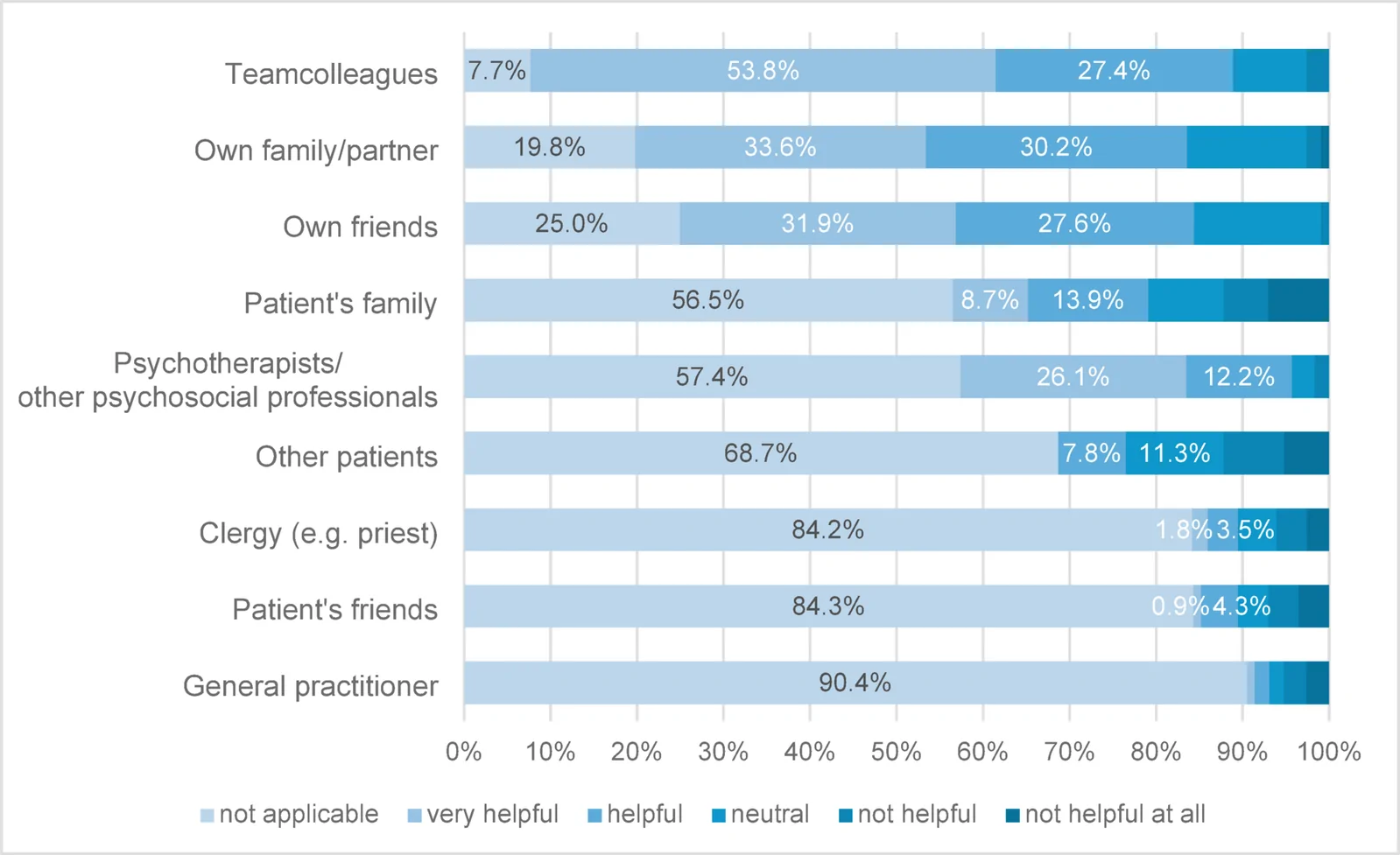
Perceived helpfulness of support offered from different groups in coping regarding the suicide. *Note*: 5-point Likert scale from 1="not helpful at all” to 5="very helpful” and an additional category “I don’t know,” with response frequencies in percentages (%), *n* = 117.

Respondents reported having sought help from a psychotherapist or other psychosocial professionals (Fig. 2) and found this support to be almost exclusively positive. Support offers from clergy (e.g., a priest) or a general practitioner were rarely used. The support from their psychotherapists received similar assessments. Most practitioners turned to their own team colleagues and rated this type of support as predominantly very helpful (53.8%) or helpful (27.4%). Family and partners were also seen as supportive. Respondents also often turned to their friends and found this support similarly helpful to that from their family and/or partner. Additionally, no one reported experiencing the contact with fellow patients as very helpful, only 7.8% chose “helpful”.

### Short-term (acute) and long-term emotional impacts of suicide

The highest scores were for feelings such as shock (M = 4.98, SD = 1.44), grief (M = 4.69, SD = 1.33), disbelief (M = 4.60, SD = 1.55), with the mean corresponding to the category „true” (see Table 4). Agreement was somewhat lower regarding feelings of depression, failure, guilt, incompetence, and discouragement, with mean values ranging from 3.69 to 2.97. However, when looking at the frequencies, it becomes clear that the values showed a large spread. Feelings that respondents generally perceived as less pronounced were anger (M = 2.47, SD = 2.05), shame (M = 2.44, SD = 1.94), and anxiety (M = 2.41, SD = 1.92). Relief had by far the lowest score among all feelings (M = 0.63, SD = 1.25).

**Table 4:** Short-term emotional impacts of suicide (*n* = 117).

Item	M	SD
Shock	4.98	1.44
Grief	4.69	1.33
Disbelief	4.60	1.55
Failiure	3.69	1.66
Depression	3.56	1.78
Guilt	3.40	1.79
Incompetence	3.27	1.89
Discoragement	2.97	1.86
Anger	2.47	2.05
Shame	2.44	1.94
Anxiety	2.41	1.92
Relief	0.63	1.25

0 = “not at all true”, 1 = “not true”, 2 = “rather not true”, 3 = “neutral”, 4 = “rather true”, 5 = “true”, 6 = “completely true”.

The highest scores were for the item “increased sensitivity to the signs of suicide risk” (M = 4.25 SD = 1.25; Table 5). Respondents also scored quite high on anxiety when assessing suicidal patients (M = 3.34, SD = 1.78). Similarly, the item asking whether psychotherapists had the impression of assessing a larger number of patients as high-risk since the suicide had a mean score of 3.43 (SD = 1.57). The following items had mean scores between 2.66 and 2.18, corresponding to “neutral” to “rather not true”: increased acceptance of suicidal behaviors, increasing concern regarding one’s own competence, repeated thoughts about patients’ suicidal behaviors, and heightened feelings of helplessness in treating suicidal patients. The item “persistent guilt feelings” had the lowest mean score (M = 1.72, SD = 1.60).

**Table 5:** Longt-term emotional impacts of suicide (*n* = 117).

Item	M	SD
Increased sensitivity to signs of suicide risk	4.25	1.25
Assessment of more patients as risk patients	3.43	1.57
Greater anxiety when assessing suicidal patients	3.34	1.78
Repeated thoughts of suicidal behavior of patients	2.72	1.74
Increased feeling of helplessness when treating suicidal patients	2.66	1.73
Increasing concerns about one’s own competence	2.57	1.90
Increased acceptance of suicidal behavior in patients	2.48	1.64
Reduced sense of one’s own effectiveness as a therapist	2.31	1.62
Reduced feeling of effectiveness of psychotherapy	2.18	1.73
Persistent feelings of guilt	1.72	1.60

0 = "not at all true", 1 = "not true", 2 = "rather not true", 3 = "neutral", 4 = "rather true", 5 = "true", 6 = "completely true"

### Impact-of-event scale - revised

The mean scores on the individual subscales for the two measurement conditions—retrospective assessment immediately after the suicide (T0) and current assessment (T1)—differ significantly (Table 6): On the Avoidance Scale, the mean score for the retrospective assessment immediately after the suicide was 6.45 (SD = 6.60), whereas the mean score for the current assessment was only 3.64 (SD = 5.99; t_(106)_ = 6.50, *p* <.001). Similarly, the scale scores for the two variants differed on the “Hyperarousal” subscale (t_(106)_ = 5.69, *p* <.001) and on the “Intrusion” subscale (t_(106)_ = 8.13, *p* <.001). The same was true for the total scores: score calculated using the formula by Maercker and Schützwohl^14,15^(t_(101)_ = 6.25, *p* <.001) and raw score (t_(101)_ = 8,49, *p* <.001). The effect size Cohen’s d was lowest for the hyperarousal scale compared to the other subscales (d = 0.55). The effect size for the avoidance scale was somewhat higher at 0.64, but the intrusion scale showed an even higher value of d = 0.79. The effect size for the total scores was about d = 0.61 resp. d = 0.84.

**Table 6:** Comparison of the IES subscale scores and the total scores at the two measurement options.

Scale	T0 retrospective		T1 current					
	M	SD	M	SD	t	df	*p*	Cohnen‘s d
Hyperarousal	7.25	8.64	3.64	6.75	5.69	106	< 0.001	0.55
Intrusion	11.09	7.64	5.51	7.06	8.13	106	< 0.001	0.79
Avoidance	6.45	6.60	3.49	5.99	6.50	104	< 0.001	0.64
IES-R raw score scale	25.80	19.17	12.84	17.56	8.49	101	< 0.001	0,84
IES-R total score*	−2.99	1.46	−3.65	1.24	6.25	101	< 0.001	0.62

M = Mean, SD = Standard Deviation, T_0_/T_1_ Measurement options, t = Test size t-test for paired samples (Degrees of Freedom), p = Probability, Cohen’s d= Effect size (Cohen, 1972); ; * Formula Maercker & Schützwohl^10,11^

As described in the sample characteristics, approximately two-thirds of the professionals surveyed had directly treated patients who had died by suicide, while about one-third had been confronted with such cases as part of their therapeutic teams. The analysis examined whether these two groups differed in terms of their IES scale scores. They did not differ, neither in the retrospective view (M_direct_ = −2.85, SD = 1.61, M_Team_ = −3.26, SD = 1.04, T = 1.36, *p* =.174) nor in the current (M_direct_ = −3.59, SD = 1.35, M_Team_ = −3.79, SD = 0.87, T = 0.81, *p* =.418).

### Explorative qualitative content analysis

The smallest unit of the qualitative analysis was individual words, while the largest units were the entire response to an open-ended question. Definitions of individual categories and corresponding coding rules for distinguishing between them can be found in the supplementary information. Overall, five main categories were identified, including “controllability of events” “emotional reactions”, “interaction and relationship”, “violent/horrific suicide” and “resources and coping strategies”. The main category “emotional reactions,” which pertains to the emotions of the psychotherapists, was divided into a total of seven subcategories during the analysis process such as grief, self-doubt, anger toward the patient, feelings of guilt and helplessness. The category “violent/horrific suicide” could not be further subdivided in terms of content, nor could it be clearly classified into the other main categories and therefore constitutes a separate main category. The subcategories “unpredictability”, “patient-therapist relationship”, and “burden on social environment” were the most frequently found in the analysis units. For example, one of the respondents expressed: *“During high-frequency psychological treatment. He was a high-risk candidate*,* but that he would take his own life during treatment and with a good therapeutic alliance was terrible.”* (Respondent No. 56). One other “*I was there for my colleagues*,* but had to talk to the other patients with the support of the ward nurse; the partner I’d had during previous stays in one-on-one care also came and fell into my arms*,* needing comfort.” („burden on social environment“*; respondent no. 143). Inexperience (“inexperience/first suicide”) was mostly directly mentioned, especially when it was the first patient suicide. Analysis units assigned to the category “feelings of guilt” were often more complex, and the feelings of guilt and “self-doubt” were predominantly expressed indirectly. For example, one participant wrote: *“The patient was very evasive - I wondered if I shouldn’t have believed her when she said she had distanced herself from the idea of suicide.“* (respondent no.15) Categories such as “identification with the patient,” and “anger at the patient” were very rare.

## Discussion

Interestingly, the preventability and predictability of patient suicides were assessed differently depending on whether the general views were solicited or if the question specifically referred to the “most distressing” suicide. Generally, a very small proportion considered suicides to be completely unavoidable or unpredictable. However, in both cases, about one-third of respondents believed that the suicide they had witnessed was entirely unpreventable or entirely unpredictable. These results align with findings from studies that also focused on the perceived predictability and preventability of patient suicides. For instance, it was shown that 32.8% of psychiatrists regarded the suicide they found most distressing as completely unpredictable and 44.8% as completely unpreventable^20^. In contrast, Eagles and colleagues^20^ demonstrated that the numbers were significantly lower when assessing the predictability or preventability of suicides in general: 9.4% believed suicides were generally unpredictable and 6.4% considered them generally unpreventable. One hypothesis that might explain these differences between the general and specific assessments of preventability and predictability is that massive feelings of guilt and self-reproach could arise if caregivers believed they could have foreseen or prevented the suicide. Eagles and colleagues^20^ also discuss this potential explanatory approach in their work. Furthermore, this might lead caregivers to lose faith in the effectiveness of psychotherapy^21^.

Overall, the literature indicates that guilt and self-reproach, anger, shock, fear, concern, loss of self-confidence, and feelings of incompetence or failure are among the most common reactions reported by caregivers after a patient suicide^22^. The most common feelings after the suicide in this sample were shock, grief, disbelief and depression. A systematic review by Sandford and colleagues^23^ summarizes the current state of research on the effects of a patient´s suicide and includes, as one area, the personal (emotional) well-being of mental health practitioners. The review seems to confirm the results of this study since most common personal reactions found were guilt, blame, shock, anger, sadness, anxiety and grief. Additionally, suicides, unlike other types of death, involve unique aspects, such as the rejection of the caregivers’ supportive efforts by the individual or the “one-sided farewell”^16,24^, which could account for the high grief scores. The scores for guilt in this sample, as well as in the sample studied by Kleespies and colleagues^16^, were similar, with M = 3.23 and M = 3.5, respectively, and were overall quite high compared to other variables. The standard deviation suggests significant individual differences in respondents’ answers.

As described, some items of the questionnaire for long-term emotional impacts had quite high values, such as “increased sensitivity to signs of suicide risk” and “greater anxiety when assessing suicidal patients”. However, it is notable that the standard deviation of this item is quite high, indicating significant individual differences among respondents in both samples. Differences in response behavior may be influenced by various factors. One potential factor is the connection between long-term emotional impacts and identity, as studies show that female caregivers are more likely to question their professional competence and suffer from self-doubt following a patient suicide than their male colleagues^25^. Gender differences in the current study were explored by Lehmann et al.^26^ who found that female psychotherapists had significantly higher IES scores than their male colleagues. A meta-analysis investigating gender-specific effects in medical communication found that women conduct significantly more psychosocial counseling and emotionally focused conversations^27^. Consequently, women working in these professions might be more acutely aware of potential misunderstandings in their conversations with the suicide victim in retrospect than their male colleagues^28^. This could negatively impact the emotional effects of the patient’s suicide and amplify concerns about their competence. An interesting finding documented in a review by MacGarry and colleagues^29^ concerns a phenomenon referred to as professional growth following exposure to patient suicide (analogous to the well-established concept of post-traumatic growth). Thus, it appears that experiencing such a crisis may lead either to psychological distress and impairment or to personal and professional development. This raises the question of which interventions following a patient suicide (postventions) may facilitate adaptive coping and positive outcomes.

Respondents who experienced a completed patient suicide scored significantly higher on the intrusion (M = 16.7, SD = 7.5) and avoidance (M = 12.1, SD = 7.6) subscales immediately after the suicide in the study conducted by Kleespies et al.^16^. However, at the time of participation in this study (T1), the mean values were more similar to those found in the study conducted by Kleespies and colleagues^16^. The differences in subscale mean scores at the retrospective measurement optionT0 between both studies might be attributed to the composition of the respective samples. Kleespies et al.^16^ surveyed practitioners still in training and with less professional experience. As discussed regarding long-term emotional impacts, lower professional experience could also relate to a more intense stress reaction measured by the IES. A large-scale study surveyed 666 psychosocial professionals (including nurses, social workers, psychiatrists, and psychologists) and used the revised version of the IES^30^. This allowed for the inclusion of hyperarousal as an additional subscale. Castelli Dransart and colleagues^29^ identified subgroups with significantly different subscale mean values through cluster analysis. The authors distinguished several “low-impact” groups, a “moderate-impact” group, and a “high-impact” group. The “low-impact” groups had significantly lower values compared to the “high-impact” group. The “high-impact” group was described as “unsupported professionals,” having reported particularly little support. Similarly, in this study, some respondents with high overall scores reported receiving no support from family or partners or did not participate in supervision. Lack of support could thus be associated with high scores on the intrusion, hyperarousal, and avoidance scales. The results are largely consistent with those in the literature, although some findings are mixed^30^. This study did not aim to determine whether respondents suffer from PTSD symptoms following a patient suicide. Nevertheless, it raises the question of whether some practitioners perceive the event as potentially traumatic. Criteria for PTSD under ICD-11^31^, such as (I) re-experiencing the traumatic event in the present accompanied by strong emotions or (II) avoidance of thoughts and memories of the event can be met by some practitioners here. However, these symptoms were clinically relevant in only a few individuals, with three practitioners scoring above the cut-off value immediately after the suicide (4% of the sample with a total score > 0). An American study found evidence of clinically significant trauma-related disorders in 3.8% of psychiatrists and 9.7% of psychiatric trainees following the suicide of a patient^32^. Similar to the present study, the authors assessed trauma-related symptoms using the Impact of Event Scale–Revised (IES-R). Regarding a suitable cut-off value for the IES-R, there are various recommendations in the literature, and no consensus exists on a value that reliably predicts the presence of PTSD^33^.

The insights gained from the qualitative content analysis support and complement the previously discussed results. Some categories represent constructs not captured by the questionnaires on short- and long-term emotional impacts or the IES-R. The categories “Patient-Therapist Relationship,” “Unpredictability,” “Inexperience (first suicide),” and “Violent/Horrific Suicide” dominated the responses. According to the expanded Criterion A in ICD-11^31^, this criterion is also met if individuals witness a threatened or actual injury, or a sudden, unexpected, or violent death of another person is also classified as a potentially traumatic event^34^. Some respondents described the suicide as horrific or violent and had long-standing or strong therapeutic relationships with the patient. The aspect of unpredictability, explicitly linked to potentially traumatic experiences in ICD-11, was also frequently identified through qualitative analysis. These results support the hypothesis that experiencing a patient suicide can have significant impacts on the mental well-being of therapists, particularly when considering the high subscale mean values of the IES-R. The categories above were also commonly coded among respondents with very high IES total scores. The subcategory “Inexperience (first suicide)” falls under the broader category “Resources and Coping Strategies.” Its frequent mention suggests that therapists might benefit from more comprehensive training on handling suicidal patients during their education^16^.

The results of this study indicate that various forms of support and interventions are evaluated differently regarding their helpfulness in coping with a suicide. Peer supervision was the most frequently used and generally considered very beneficial in processing the event. The same applies to formal supervision and case discussions. The qualitative analysis suggests that formal supervision was often not offered, leading respondents to feel unsupported. The exchange with colleagues was also considered very helpful, aligning well with the generally positive evaluation of peer supervision. Overall, collegial exchange seems to positively impact coping with a patient’s suicide by reducing feelings of isolation and burden^21^. However, in some cases, severe feelings of shame towards colleagues can make such exchanges counterproductive for functional coping with the suicide^35^. Additionally, personal friends, family, and partners were perceived as significant supports, although about a quarter of respondents indicated they did not seek support from these sources.

In this study’s sample, a few therapists rated supervision as not helpful or even entirely unhelpful. In cases where supervisors reassured therapists that they had acted appropriately with the suicidal patient or shared some responsibility for the suicide, this could positively impact coping with the event^35^. According to earlier research, supervision was deemed unhelpful when therapists were encouraged to talk about the suicide too soon or when supervisors immediately shared their own experiences^36^. These findings suggest that merely having structures in place to support coping may not be sufficient; the quality and implementation of these structures also play a crucial role. In this context, Cummings^37^ recently published findings from a Delphi consensus study. The experts surveyed emphasized that, rather than implementing standardized solutions, individualized support approaches are needed. The focus should be on emotional support that fosters supportive and constructive communication among colleagues across hierarchical levels.

## Limitations

During the analysis of the IES-R, it became evident that there are significant differences in scaling, cut-off values, and corresponding interpretations between various language versions of the questionnaire. This considerably limits the comparability of total scores presented in different studies. Additionally, using an instrument that measures PTSD symptoms may not be sufficient to fully capture psychological distress, as depression, for example, might also play a role. Furthermore, it should be noted that at the time of the survey as well as at the retrospective measurement option (T_0_), other stress factors in the therapists’ lives could have increased vulnerability or led to moderation effects. Another methodological limitation is the nature of the data collection. It is based on self-reporting; incorporating other perspectives, such as those of partners, colleagues, or family members, would likely provide a more accurate depiction of the actual burden. Retrospective surveys also pose a significant risk of distorted perception, as memory is influenced by complex processes. When participants are asked retrospectively to rate how distressing an event was, there is a risk of recall bias: retrospective evaluations do not necessarily reflect the level of distress that was actually experienced at the time. Retrospective reports may be systematically biased. One of the most robust findings in this context is the *Fading Affect Bias (FAB)*. FAB describes the phenomenon that, as the temporal distance from an event increases, the perceived distress associated with it is often underestimated. This occurs because the emotional intensity of negative memories tends to fade more rapidly, on average, than that of positive memories. While the factual details of an event may remain well remembered, the associated negative affect diminishes to a greater extent. However, this effect does not apply equally to all individuals, which is particularly relevant for the present sample. FAB has been shown to be a robust phenomenon in healthy individuals^38^. In contrast, the effect appears to be altered in individuals with mental disorders. Individuals with depression frequently exhibit a *negativity bias*, whereby negative memories remain more emotionally vivid and the associated emotions decline less over time than they do in healthy individuals^39^. In the case of highly significant or traumatic events, disorder-specific cognitive and emotional processes may even reverse the direction of the Fading Affect Bias^40^. Although no formal clinical assessment was conducted in the present study, the response patterns suggest that, in addition to psychologically healthy participants, the sample may also include individuals with symptoms indicative of depression or post-traumatic stress disorder (PTSD). However, the sample size is too small to permit meaningful post hoc subgroup analyses examining differential patterns of the Fading Affect Bias. Another source of recall bias arises from participants’ current psychological state, which can substantially influence retrospective evaluations of past events. Therapists who continue to experience psychological distress or trauma may recall the event as having been more distressing than it was immediately following the suicide, a phenomenon referred to as *mood-congruent recall*. Conversely, therapists who have successfully processed the event may underestimate the distress they experienced at the time or may remember it in less detail. In the present sample, participants did not share a comparable level of current psychological well-being, making this a potential confounding factor.

## Conclusions

In summary, it can be assumed that a patient’s suicide has both acute and long-term emotional impacts on the respondents. Intense feelings of grief, bewilderment, and shock play a predominant role. Additionally, the suicide leads therapists to feel anxious about assessing suicidal patients. Both the short-term and long-term emotional impacts seem to be related to views on the predictability and preventability of suicides in general, as well as regarding the experienced suicide. The partially high scores on the IES-R subscales indicate that the suicide results in similar psychological distress as other potentially traumatic experiences. This is also supported by the qualitative analysis, which identified the most distressing aspects as the patient-therapist relationship, the unpredictability of the suicide, the inexperience (i.e., limited professional experience in dealing with suicidality), and the violent/horrific nature of the suicide.

Overall, the literature reports mixed findings regarding situational and individual characteristics that influence the impact of suicide on the therapist’s mental health. Most therapists do not develop clinically significant psychopathology. Nevertheless, there are also individuals who suffer significantly from this event. Even in cases where the psychological distress is very pronounced immediately after experiencing a patient suicide, most respondents observe symptom improvement over time. Factors likely related to the extent of emotional impacts include years of professional experience and the therapist’s relationship with the patient, as a perceived close relationship seems to influence emotional distress. Since previous work has focused on sociodemographic variables and characteristics, it might be necessary to examine relationship aspects more closely to better understand the connection. The current analysis of qualitative and quantitative data could not definitively determine if the therapists which IES-R scores were identified as particularly high share common risk factors. Further qualitative research might provide insights to derive findings for prevention and postvention for those individuals. This may also be important for preventing further suicidal behavior in patients, as distress or conflicting emotional responses by clinicians can, in turn, have a significant impact on the therapist-patient interaction^7^. In our view, this should also be incorporated more fully into the training of future psychotherapists.

## Supplementary Information

Below is the link to the electronic supplementary material.

Supplementary Material 1

## Data Availability

The data that support the findings of this study are available from the corresponding author upon request.
