# Supplementary material for: The impact of patients suicide on the mental health of psychotherapists
Source: Sci Rep. 2026 Sep 3;16:27656. doi: 10.1038/s41598-026-67919-7 (PMC13542113; doi:10.1038/s41598-026-67919-7)
Supplement: Supplementary file 1 [file 41598_2026_67919_MOESM1_ESM.pdf]

Supplementary Material

Table 1: Category System with Definitions, Anchor Examples, and Coding Rules

**Main Category**	**Subcategory**	**Definition**	**Anchor Example**	**Coding Rules**
1. Emotional Response	1. Helplessness	Helplessness refers to the feeling that a person has no control over a situation or is not able to protect themselves or others or solve their problems. It is a state of powerlessness and lack of action options, often associated with fear, frustration, and despair.	*"The patient seemed very desperate and therefore repeatedly sought out the clinic, yet we found no glimmer of hope to work on"* (R. no. 141)	The feeling that a person has no control over a situation or is not able to protect themselves or others or solve their problems is crucial.
	1.2 Compassion	Compassion is an empathetic reaction to the suffering of others. It involves understanding the feelings and needs of other people, as well as the desire to help or support them in difficult situations.	*"And I believe it got to me because I admired how she managed the most difficult life circumstances, her adulthood, and her family relationships so well."* (R. no. 98)	Compassion is to be distinguished from grief by expressing concern about the situation of another person. It is not primarily about one's own negative affect.
	1.3 Guilt	Guilt is an emotional reaction that arises when a person feels they have committed a wrongdoing against themselves or others, or are responsible for a negative event.	*"This regret, these guilt feelings occupy me."* (R. no. 50)	Guilt feelings are either directly named or implied, for example, when the caregiver believes they did not act correctly towards the patient or feel responsible for the suicide.

Main Category	Subcategory	Definition	Anchor Example	Coding Rules
	1.4 Anger at the Patient	Anger is an intense emotion that arises in response to a perceived threat, frustration, injustice, or provocation. In this case, it is directed towards the patient or the suicide victim.	*"Anger at the patient after suicide"* (R. no. 113)	Anger is directly identified as a feeling to allow for a clear distinction from the category of "unfulfilled hopes."
	1.5 Grief	Grief refers to a complex emotional reaction due to the loss of a person.	*I was shocked and saddened after the wife's phone call"* (R. no. 117)	Grief should be the primary feeling and should be directly identified by the participant.
	1.6 Self-doubt	Self-doubt refers to the feeling of uncertainty or lack of conviction regarding one's own abilities, worth, or self-image. People with self-doubt may have a fear of failure or rejection. Self-doubt can impair self-confidence and self-esteem.	*"Was the timing assessed correctly, and should the monitoring accordingly have been more stringent? The question remains unanswered concerning one's own objectivity."*(R. no. 35)	Self-doubts regarding one's own actions in connection with the suicide are directly named or hinted at. In this context, doubts about one's own abilities should be emphasized to clearly distinguish them from feelings of guilt.
	1.8 Unfulfilled Hopes	"Unfulfilled hopes" refers to situations in which a person has hopes, wishes, or expectations that are not met. It describes the state in which someone hoped for or relied on certain events to occur, but these hopes are not realized.	*"I simply wished very much for her to make it. She had also improved, was close to being discharged."* (R. no. 26)	It becomes clear that there was a positive expectation towards the patient and the therapy process, which, however, was not fulfilled. Grief or regret and compassion are secondary.

**Main Category**	**Subcategory**	**Definition**	**Anchor Example**	**Coding Rules**
2. Interaction and Relation-ship	2.1 Burden Social Environ-ment	Intense emotional reactions from the patient's social environment (such as family and friends), in this case, due to the suicide of the patient can cause the therapist to experience emotional stress, psychological pressure or strain.	*"I was there for my colleagues, had to speak with other patients with the support of the attending physician, the partner whom I had individual contact with during previous admissions also came and fell into my arms needing comfort."* (PB no. 143)	Explicit stressors affecting persons close to the patient are identified or described, indicating that individuals in contact with the patient were burdened.
	2.2 Patient-Therapist-Relationship	„Patient-therapist relationship" refers to the interaction and connection between a patient and their therapist in a therapeutic setting. This relationship is crucial for the success of the therapy or treatment and is based on trust, respect, openness, and understanding.	*"From my side, the trust relationship was called into question by the suicide"* (R. no. 113)	The therapist identifies aspects of the interpersonal relationship with the patient.
	2.3 Identification with the Patient	"Identifying with someone" means that a person establishes a connection with another person by perceiving commonalities in experiences, feelings, attitudes, or life circumstances.	*"Identification with the patient (I am also gifted like he was)*." (R. no. 63)	The therapist describes parallels or commonalities between themselves and the patient.

Main Category	Subcategory	Definition	Anchor Example	Coding Rules
3. Controllability of Events	3.1 Unavoidability	Unavoidability refers to the characteristic of a situation or event that cannot be prevented or avoided. It implies that something is unavoidable, regardless of efforts to prevent or change it.	*"After consulting with the ethics committee, the management, and the department head, we decided to end the prolonged fixation and treat the patient in a 'secured' single room without strict isolation but with reduced furniture ('firm' mattress, indestructible blanket) and in a so-called 'secure' gown in this room with high frequency.“* (R. no. 150)	It is described that attempts to act were made, but the suicide could not be prevented.
	3.2 Avoidability	Avoidability refers to the ability to prevent or circumvent something. It means that a situation, event, or problem can be avoided through appropriate measures or actions. The avoidability can depend on various factors, including the availability of resources, the presence of knowledge and the assessment of risks and consequences.	*"On the other hand, I believe it is possible that it was an impulsive act that could have been avoidable or postponable with better available support."* (R. no. 156)	According to the therapist, there could have been possibilities to potentially prevent/avoid the suicide.

Main Category	Subcategory	Definition	Anchor Example	Coding Rules
	3.3 Unpredictability	Unpredictabi-lity describes the characteristics of suicides that were difficult or impossible to foresee in advance. This unpredictability can lead to uncertainty, confusion, and stress, as it can impair the sense of control or planning.	"Partial inpatient treatment was planned; during the initial interview, the patient credibly distanced themselves from acute suicidality, there were no indications of self-harm or harm to others, they were emotionally stable" (R. no. 45)	Unpredictability is clearly identified or described. To distinguish it from unavoidability, it is important to note that there were no signs, and accordingly, no attempts to act were made to prevent such an event.
	3.4 Predictability	Predictability refers to the characteristic of suicides that could have been predicted or recognized in advance. It means having a certain expectation or assessment of how something will develop based on known factors, patterns, or experiences.	*"The patient was repeatedly suicidal in phases, but always able to make agreements. Recently, however, there was great resignation in the patient and no longer possible to distance themselves from it.“* (R. no. 139)	Signs of suicidality were described, or predictability was mentioned. Feelings of helplessness, if present, must be secondary.
	3.5 Assigning Blame	Blame assignments refer to the act of transferring responsibility for a negative event or mistake to another person or group of people. It is a process of blaming others to relieve oneself of responsibility.	*"Accusations against the staff by one of the patient's fellow patients“* (R. no. 113)	It is described that the treatment team was blamed for the suicide.

Main Category	Subcategory	Definition	Anchor Example	Coding Rules
4. Resources and Coping Strategies	4.1 Inexperience (First Suicide)	Inexperience refers to a lack of experience or knowledge in a specific area. It can mean that someone has not yet gained enough experience to effectively handle certain tasks or challenges.	*"It was also the first suicide I experienced in the course of my work in psychiatry."* (R. no. 26)	Inexperience in dealing with suicides or inexperience in general was the focus.
	4.2 Lack of Support	"Lack of support" describes a situation where someone does not receive the help, encouragement, or support they need or expect. This can relate to various areas of life, including personal relationships, professional environment or social support.	*"I was on the verge of burnout. There was no relieving team supervision."* (R. no. 105)	It was described that offers of help or support for coping were missing.
5. Violent / Cruel Suicide		A suicide perceived as cruel refers to a self-inflicted death that is seen as particularly shocking, tragic, or disturbing due to its method or circumstances. This can include a variety of factors, such as choosing a particularly violent or publicly visible method.	*"I also found the manner of the suicide (hanged in the shower) to be horrifying. The thought of the moment until she is dead, I believe that is the most dreadful part of all suicides."* (R. no. 105)	The characteristics of the suicide and the emotional impact on the caregivers were described.
